# Epidemiologic and evolutionary relationships between Romanian and Brazilian HIV-subtype F strains.

**DOI:** 10.3201/eid0103.950305

**Published:** 1995

**Authors:** C I Bandea, A Ramos, D Pieniazek, R Pascu, A Tanuri, G Schochetman, M A Rayfield

**Affiliations:** National Center for Infectious Diseases, Centers for Disease Control and Prevention, Atlanta, Georgia, USA.


### Epidemiologic and Evolutionary Relationships between Romanian and Brazilian HIV-1 Subtype F Strains

The initial classification of HIV-1 viruses as Western or African strains has been replaced by phylogenetic subtyping that uses nucleotide sequence data. Eight distinct phylogenetic HIV-1 lineages or subtypes, A to H, have been defined (1). Considering that the rate of HIV-1 genome evolution is estimated at 0.5% to 1% per year and that the average genetic distance between the HIV-1 subtypes is approximately 20%, it is likely that these subtypes originated before the HIV-1 pandemic (1). The global mosaic of HIV-1 subtypes is consistent with the hypothesis that most regional epidemics started with the introduction of one or a few variants that diversified locally rather than through radiant waves of already diversified HIV-1 subtypes that spread from the place of origin. In this report, we address the evolutionary and epidemiologic relationships between the HIV-1 subtype F viruses recently identified in two geographically distinct regions, Romania and Brazil.

The HIV-1 epidemic among Romanian children living in orphanages was recognized during 1989-1990 (2). Epidemiologic studies have shown that most children became infected by horizontal transmission of HIV-1 through blood transfusions or through the use of unsterilized medical equipment. We have shown by nucleotide sequence analysis that all HIV-1 isolates from children in southeastern Romania are highly related genetically (3). The average interperson nucleotide distance within the C2-V3 region of the *env* gene was 0.9% to 3.6%. Phylogenetic analysis of these sequences showed that the Romanian HIV-1 strains clustered together with a single Brazilian HIV-1 strain in a previously unrecognized evolutionary clade later designated as the F subtype. We now have the opportunity to augment this comparison with additional F subtype sequences from the two countries and to further address the relationship between Romanian and Brazilian viruses.

In the phylogenetic analysis of the envelope C2-V3 nucleotide sequences (Figure 1), we included eight HIV-1 F subtype viruses isolated from children in southeastern Romania (L19570-L119579) (3) as well as two representative sequences of the HIV-1 viruses found in children living in northcentral Romania (R18586 and R18598) (Bandea et al., manuscript in preparation). From Brazil, we included, in addition to the sequence of the first identified F subtype strain (BRA7944)(4), three recently reported F sequences (BZ126A, BZ162A, and BZ163A) (5) and four F strains (BR46, BR57, BR58, and BR59) isolated in our laboratory from patients in Rio de Janeiro. We also included two HIV-1 strains from Cameroon, CA4 and CA20, that have been tentatively classified as F subtype viruses (1,6) and reference nucleotide sequences representing the other HIV-1 subtypes.

The results of this phylogenetic analysis show that the Brazilian and Romanian sequences cluster in two highly related but separate groups. The genetic distance between Brazilian subtype F sequences was 5% to 13.8% , which is within the limits of the established intrasubtype distance values (1). Among Romanian C2-V3 nucleotide sequences this distance was 0.9% to 6.5%, and between the two groups it was 7.5% to 12.9%. These values support the inclusion of the Romanian and Brazilian groups within the same F HIV-1 subtype. The reliability of these phylogenetic results was verified by bootstrap analysis (100 data sets) and by pruning, which consists of sequential removing of different strains and rerunning the analysis.

The two sequences from Cameroon associated only weakly with Romanian and Brazilian groups, and this association was not stable. The genetic distance between the Romanian and Brazilian sequences and sequences from Cameroon was 16.5% to 24.1%, which is typical of intersubtype rather than intrasubtype genetic distances. Our analysis does not support a strong linkage between the Cameroonian strains and the other F subtype viruses; however, in evolutionary terms, these viruses may be closer to each other than to the other subtypes, or they may have undergone convergent evolution within the envelope region that we analyzed. Analysis of additional sequences from other regions of the HIV-1 genome may clarify the relationship between the Cameroonian strains and the other subtypes.

The amino acid GPGR motif at the tip of the V3 protein loop has been considered a signature sequence for the Brazilian F subtype viruses (5). This relatively conserved motif was noticeable because it is characteristic among B subtype viruses, whereas the GPGQ sequence predominates among all the other HIV-1 strains, including all the initial Romanian F viruses. Two of the newly identified Brazilian strains (BR58 and BR59), however, contain the GPGQ motif, and the Romanian strain R18598 contains the GPGH motif (Figure 2). Phylogenetically, these sequences group with their respective geographic clusters (Figure 1), which indicates that independent mutations may have occurred at this locus.

As indicated earlier, the genetic distance between Romanian HIV-1 nucleotide sequences is very small, which suggests a direct epidemiologic link among these strains and a short period of evolution. The exclusive presence of highly related viruses in two geographically distinct provinces of Romania strongly suggests that the initial pediatric HIV-1 epidemic in this country started from a single infectious source. This suggestion is also supported by the fact that F subtype viruses are uncommon and, therefore, the chances for independent multiple introduction of highly related F subtype strains in Romanian children are very low. Although no nucleotide sequence data are available about HIV-1 strains circulating in the adult population in Romania, the epidemiologic and serologic studies indicate that the number of infections is small, and probably most HIV-1-infected adults were infected though sexual contact with foreign visitors or with Romanians that traveled outside the country (2). It is expected, therefore, that most of the HIV-1 strains infecting the adults represent internationally prevalent subtypes. It is remotely possible, however, that adults were infected with F subtype viruses and could have served as the original or intermediary carriers for HIV-1 transmission among the groups of children living in distinct geographic regions of Romania.

In Brazil, the relatively long genetic distance between F subtype viruses could indicate that a single ancestor was introduced during the early phases of the HIV-1 epidemic and diverged locally, or that multiple different F strains were introduced to this country. The low prevalence of F subtype viruses worldwide makes the latter alternative less likely. However, no information is available about the HIV-1 strains present during the early phases of the epidemic in Brazil. Our ongoing studies and the published data (4,5) indicate that the HIV-1 F subtype infections represent roughly 10% of the estimated number of cases. This relatively large number of F subtype infections and the estimated rate of HIV-1 divergence suggest an early introduction of the F subtype viruses in Brazil.

Because of the geographic position and the sparsity of socioeconomic relations between Romania and Brazil, the potential for an epidemiologic link between HIV-1 F subtype viruses is small. A more compelling argument against a direct epidemiologic link between F subtype viruses from these two countries can be made on the basis of the topology of the phylogenetic branches that set apart the two groups of viruses (Figure 1). If a direct epidemiologic link existed between the two groups, the clustering would be integral with one group branching from within the other. The tree topology shows the two groups of viruses on separate branches with a relatively distant common ancestor. Although limited in scope, our findings support an evolutionary relationship between the Romanian and Brazilian F subtype

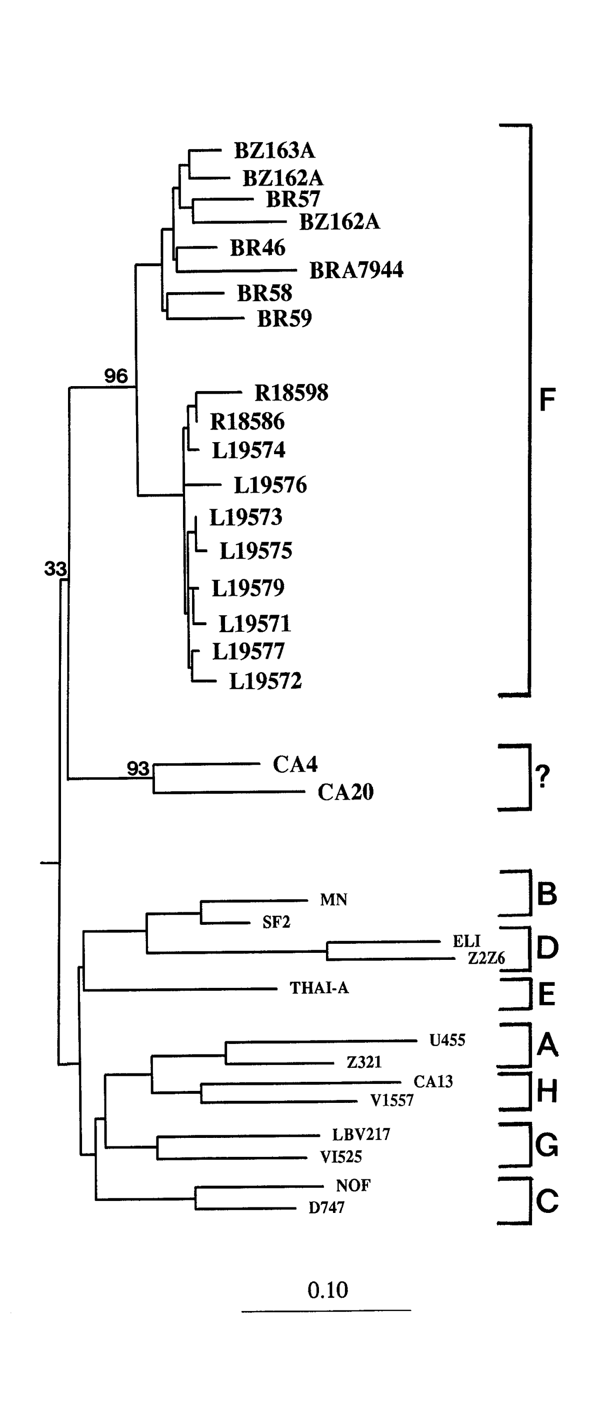

Figure 1. Phylogenetic relationship between Romanian and Brazilian subtype F nucleotide sequences. The tree was constructed by using the neighbor joining method included in the Phylip 3.5c package (7). Three hundred and two aligned nucleotides from the envelope C2-V3 region were used for analysis. The vertical distance between the branches is noninformative and for clarity only. Numbers at the branch nodes indicate bootstrap values. The nucleotide sequence distance among strains can be deduced by using the bar scale included in the figure.

Figure 2. Alignment of deduced amino acid sequences for envelope C2-V3 region of Brazilian and two representative Romanian HIV-1 F subtype strains and their comparison with Cameroonian sequences and the consensus sequences for some of the other subtypes. F CON represents consensus amino acid sequence (single letter code) for the Romanian and Brazilian F subtype HIV-1 viruses presented in this figure. Consensus sequences for the other subtypes are from Ref. 1. Amino acids identical to the F CON are shown as a dash, and the dots represent gaps introduced to align sequences. The top bar shows the peptide motif at the tip of the V3 protein loop.

viruses and indicate that the two regional epidemics arose independently.

**Claudiu I. Bandea,* Artur Ramos,*† Danuta Pieniazek,* Rodica Pascu,‡ Amilcar Tanuri,† Gerald Schochetman,* and Mark A. Rayfield***

*National Center for Infectious Diseases, Centers for Disease Control and Prevention, Atlanta, Georgia, USA; †Universidade Federal do Rio de Janeiro, Rio de Janeiro, Brazil; ‡University of Medicine and Pharmacy, Tg-Mures, Romania

### References

1. Myers G, Korber B, Wain-Hobson S, Smith R, Pavlakis G, eds. Human retroviruses and AIDS 1994: a compilation and analysis of nucleic acid and amino acid sequences. Los Alamos, NM: Los Alamos National Laboratory, 1993.
2. Hersh BS, Popovici F, Apetrei RC, et al. Acquired immunodeficiency syndrome in Romania. Lancet 1991;338:654-9.
3. Dumitrescu D, Kalish ML, Klicks SC, Bandea CI, Levy JA. Characterization of human immunodeficiency virus type 1 isolates from children in Romania: identification of a new envelope subtype. J Infect Dis 1994;169:281-8.
4. Potts KE, Kalish ML, Lott T, et al. Genetic heterogeneity of the V3 region of the HIV-1 envelope glycoprotein in Brazil. AIDS 1993; 7:1191-7.
5. Louwagie J, Delwart EL, Mullins JI, McCutchan FE, Eddy G, Burke DS. Genetic analysis of HIV-1 isolates from Brazil reveals presence of two distinct genetic subtypes. AIDS Res Hum Retroviruses 1994;10:561-7.
6. Nkengasong JN, Janssens W, Heyndrickx L, et al. Genotypic subtypes of HIV-1 in Cameroon. AIDS 1994;8:1405-12.
7. Felsenstein J. PHYLIP-phylogeny interference package (version 3.2). Cladistics 1989;5:164-6.
